# Patterns of dyslipidemia amongst hypertensive patients in Abuja, North Central Nigeria

**DOI:** 10.11604/pamj.2021.39.11.28807

**Published:** 2021-05-04

**Authors:** Henry Chijioke Onyegbutulem, Dilli Dogo, Francis Alu, Musa Dankyau, David Samuel Olorunfemi, Faruk Mustapha Abdullahi, Isaac Olubanji Akerele, Nafisah Ja'afar Bala, Ugo Nnenna Ibeabuchi, Maimuna Onyi Mohammed

**Affiliations:** 1 Department of Internal Medicine, College of Medicine and Health Sciences, Nile University of Nigeria, Asokoro District Hospital, Abuja, Nigeria,; 2 Department of Surgery, College of Medicine and Health Sciences, Nile University of Nigeria, Asokoro District Hospital, Abuja, Nigeria,; 3 Department of Obstetrics and Gynecology College of Medicine and Health Sciences, Nile University of Nigeria, Maitama District Hospital, Abuja, Nigeria,; 4 Department of Family Medicine, Bingham University, Karu, Bingham University Teaching Hospital, Jos, Nigeria,; 5 Department of Internal Medicine, Bingham University, Karu, Bingham University Teaching Hospital, Jos, Nigeria,; 6 Department of Physiology, Nile University of Nigeria, Abuja, Nigeria,; 7 Department of Family Medicine, Maitama District Hospital, Abuja, Nigeria,; 8 Department of Internal Medicine, Asokoro District Hospital, Abuja, Nigeria

**Keywords:** Pattern, dyslipidemia, hypertensive patients, Abuja

## Abstract

**Introduction:**

dyslipidemia and hypertension are independent cardiovascular risk factors that are linked by insulin resistance and commonly coexist as components of the metabolic syndrome. The consequences of dyslipidaemic patterns includes high cardiovascular risk profile and sequalae. Data on lipid patterns amongst hypertensive patients in Abuja, a city with affluent pattern of life style and rising cardiovascular risk profile is very scanty or limited to just levels of total plasma cholesterol. This study was aimed at determining the pattern and frequencies of dyslipidaemic forms among hypertensive patients in Abuja.

**Methods:**

this was a retrospective cohort study with the following data collected; socio-demographics, anthropometric measures and certain metabolic parameters from the new and old groups and analyzed using IBM SPSS Statistics, version 23. All decisions were made using the assymp. Two-sided Pearson χ2 probability. Quantitative variables were expressed as means and standard deviations. Qualitative variables were expressed as percentages. A p-value of ≤0.05 was set as significant.

**Results:**

eight hundred and fifty eight (858) cases were enrolled for this study, 704 (82.1%) olds and 154 (17.9%) news. There were 251 (29.3%) males and 607 (70.7%) females. The means of the two groups were largely comparable. The commonest dyslipidaemic type was reduced High density lipoprotein, HDL (96.1%), followed by elevated low-density lipoprotein, LDL (78.6%), then, elevated total cholesterol, (62.3%) TCHOL and elevated triglycerides, TG 43.5%.

**Conclusion:**

dyslipidemic forms are very common among hypertensive patients in Abuja, Nigeria, particularly reduced HDL. Some observed associations include; middle age, female gender, middle/upper socio-economic classes, low levels of physical activity, overweight/obesity, dysglycaemia and long-standing hypertension.

## Introduction

Dyslipidemia and hypertension are independent cardiovascular risk factors that are linked by insulin resistance and commonly coexist with other cardiovascular risk factors such as dysglycaemia and truncal obesity in a cluster as seen in metabolic syndrome [1,2]. Insulin resistance is related to lipoprotein lipase deficiency which affects lipid metabolism and consequent dyslipidemia [3]. Also, insulin resistance is associated with abnormal redistribution of fatty acids, including its influx in the liver and other abdominal viscera, producing tumor necrosis factor alpha (TNF-a), which is thought to be key in truncal obesity [4], a feature currently recognized as a constant component of the metabolic syndrome [5]. Interestingly, in animal studies, a gene, the cd36 gene which encodes fatty acid translocase, appears to underlie insulin resistance, defective fatty acid metabolism and dyslipidemia in hypertension [6]. Previous human studies had reported common patterns of dyslipidemia in hypertension as include; increased total plasma cholesterol, triglyceride and low-density lipoprotein (LDL-C) cholesterol, decreased high-density lipoprotein (HDL-C) cholesterol, changes in the composition of LDL-cholesterol viz, small dense LDL-cholesterol, and increased electronegativity of LDL-cholesterol [7,8]. These changes make LDL-cholesterol susceptible to oxidation and glycation, with foam cell formation, endothelial dysfunction and thus atherosclerosis [7-9]. Consequently, there is an increased cardiovascular disease risk, a leading cause of morbidity and mortality [10].

The consequences of dyslipidaemic patterns have been largely reported in previous studies [11-13]. Elevated total cholesterol is known to play key roles in both initiation and progression of atherosclerosis with long term clinical consequences [11]. The role of elevated LDL-C in the development of CVD cannot be overemphasized, since high levels of LDL-C not only cause atherosclerosis, pharmacological interventions that reduce LDL-C cholesterol are associated with stabilization and regression of atherosclerosis [12]. Low levels of HDL-C and high triglycerides have been consistently reported in cardiovascular disease states [13]. Plasma lipid levels vary significantly in various populations, depending on differences in geographical locations, cultural, economic and social conditions, dietary habits, genetic makeup and coexisting disease states [14] such as hypertension. Studies have severally suggested that, hypertension is independently associated with dyslipidemia among Nigerians [15,16]. Abuja, the capital city of Nigeria, one of the most expensive cities to live in Nigeria, is characterized by an affluent life-style with atherogenic dietary patterns, which may impact on the cardiovascular disease profile of its inhabitants [17]. Data on lipid patterns amongst hypertensive patients in Abuja, is very scanty or limited to total plasma cholesterol only [18]. Information on plasma lipid pattern and prevalence of dyslipidemia among patients with hypertension in Abuja, is important. The objective of this study therefore, is to determine the prevalence and pattern of dyslipidemic forms among hypertensive patients in Abuja, and its possible associations, with a view to providing data that may be useful in cardiovascular disease interventions and prevention.

## Methods

**Study site and subjects:** data was extracted from the medical records of hypertensive patients who were seen over a five-year period from January 1^st^ 2015 to December 31^st^ 2019 at the medical out-patients unit of the Asokoro District Hospital, Asokoro in Abuja, the Federal capital city, FCT, North Central Nigeria. Ethical clearance with reference FCTA/HHSS/HMB/ADH/044/20 was obtained from the Asokoro District Hospital Ethics committee. A total of one thousand and three (1003) case folders were examined but eight-hundred and fifty-eight (858) 85.6%, with sufficient records were used for the study.

**Two groups of hypertensive patient:** the group designated old is made up of those patients who were previously known to have hypertension, and already on antihypertensive drugs, with other drugs such as statins and antiplatelets. They had follow-up visits. The second group designated new, consists of those with previously undiagnosed hypertension, they were just found incidentally to have hypertension and referred to the medical out-patients services for further attention. They were drug-naïve at entry. Only case files with documented proof that the patients in the new group had their hypertension confirmed by standardized criteria [19] were selected.

**Exclusion criteria:** very scanty records, and records of patients with existing chronic kidney disease and other severe illnesses such as tuberculosis, HIV, other endocrinopathies such as hyperthyroidism and Cushing´s disease were excluded.

**Study variables, data collection and data handling:** data was extracted into an Excel spread sheet, before exporting to SPSS for analysis. Data include; socio-demographic variables such as age, sex, socioeconomic class and physical activity (high, moderate and low). Family history of hypertension and the duration of hypertension (for the olds). Anti-hypertensives were documented for this group only. This information was omitted for the new patients. Other variables were blood pressure, height, weight, waist and hip circumference, body mass index (BMI). BMI< 18.5 was recorded as underweight; 18.5-24.9 as normal; 25-29.9 as overweight; and >30 as obese [20]. Waist-hip ratio (waist circumference/hip circumference) was used as a measure of truncal obesity: >0.85 in women and >0.95 in men. Others were fasting blood glucose and four parameters of lipids profile viz total cholesterol (TCHOL), high density lipoprotein cholesterol (HDL-C), low density lipoprotein cholesterol (LDL-C) and triglyceride (TG). The outcome variable was dyslipidemia which was defined as the presence of any of high total cholesterol (TC) (>5.2 mmol/L), raised low-density lipoprotein cholesterol (LDL-C) (>3.38 mmol/L) or reduced high-density lipoprotein cholesterol (HDL-C) based on the Adult Treatment Panel III (ATP III) guidelines [21]. Age cadre: for purposes of this study, patients were classified in to three groups; less than 45 years, ≥ 45-64 and >64 years. Data was analyzed using IBM SPSS Statistics, version 23. All decisions were made using the assymp. 2-sided Pearson Chi-Square (Ï‡^2^^0^) probability value. Quantitative variables were expressed as means and standard deviations. Qualitative variables were expressed as percentages. A p-value of ≤0.05 was set as significant.

## Results

**Figure 1:**
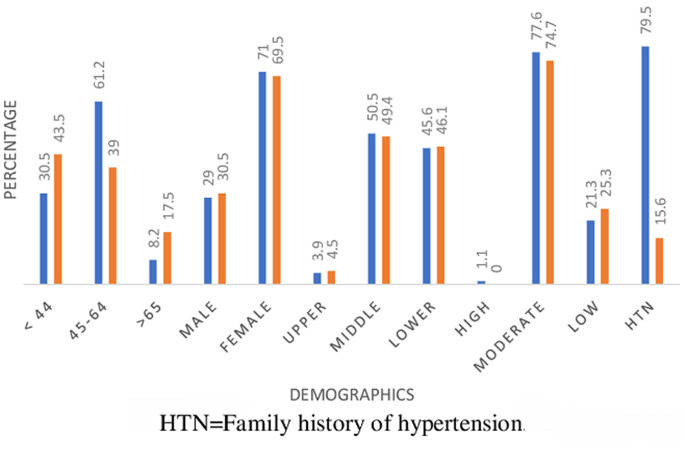
socio-demographics of the study subjects

**Figure 2:**
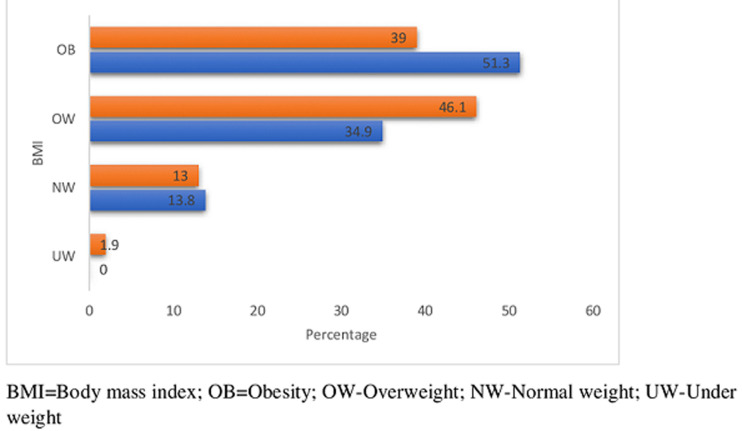
frequencies of distribution of BMI

**Figure 3:**
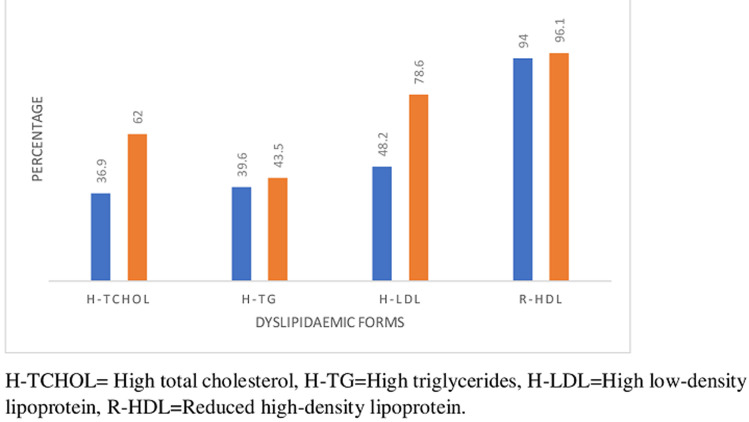
frequencies of dyslipidaemic forms

Eight hundred and fifty-eight, (858) cases were enrolled for this study, 704 (82.1%) olds and 154 (17.9%) news. There were 251 (29.3%) males and 607 (70.7%) females. Table 1 shows the means and SD of quantitative variables for the two groups. The means of most variables in both groups were comparable, although most of the lipid parameters appeared more disturbed in the new-group. Figure 1 is the frequency chart of some socio-demographics; age distribution, sex distribution, socio-economic classes (upper, middle and lower), level of physical activity, (high, moderate and low) and Family history of hypertension, HTN. The patients were mostly females in the 45-64 years age range, mostly in the middle and low socio-economic classes, with moderate levels of physical activities. Documented family history of hypertension was more frequent in the old group. Figure 2 displays the frequency distribution of categories of body mass index, BMI. Obesity and overweight were frequent in both groups and surprisingly obesity was commoner in the old group than new. Most of the patients had hypertension for a maximum of 15 years. Figure 3 displays the frequencies of the patterns of dyslipidemia. The new group had higher frequencies for all the dyslipidemias. The most frequent dyslipidaemic form was reduced HDL, seen in 96.1% and 94% of new and old respectively; followed by elevated LDL, 86% and 48.2% in new and old respectively. Figure 2 displays the relationships between the types of dyslipidemia viz elevated total cholesterol, elevated LDL, elevated triglycerides and reduced HDL, and certain variables including BMI, dysglycaemia and socio-demographics.

**Table 1:** means of quantitative variables in the old and new groups

Variables	Old-Mean+Standard deviation	New-Mean+Standard deviation
Age (years)	49.2+ 11.06	48.15 +13.25
Systolic Blood Pressure (mmHg)	151.55+ 12.96	151.77+ 11.93
Diastolic Blood Pressure(mmHg)	92.18+ 12.36	89.45 +12.48
Height (m)	1.6+0.08	1.64 +0.05
Weight (kg)	79+14.84	76.89+ 12.89
Waist Circumference (m)	98.6+13.20	98.17+ 14.97
Hip circumference (m)	110.2+ 13.19	107.4 +12.03
Body Mass Index(BMI) Kg/m2	30.8+5.91	28.7+ 6.43
Waist/Hip ratio	0.9+0.07	0.91+ 0.07
Fasting Blood Glucose (mmol/l)	5.2+1.57	4.99+ 0.82
Total Cholesterol(mmol/l)	4.95+1.10	5.73 +1.18
Triglycerides(mmol/l)	1.49+0.79	1.45 +0.69
Low Density Lipoprotein(mmol/l)	3.24+0.97	3.96 +1.04
High Density Lipoprotein(mmol/l)	1.14+0.26	1.16 +0.22
Uric Acid (mg/dl)	298.3+170.01	308.9 +160.32

**Relationships with age:** age related significantly, (p<0.05), with elevated total cholesterol in those less than 65 years in the new group, and in all age categories in the old group. The relationship was not significant (p=0.267), in those older than 65 years in the new group. The relationship with elevated LDL was not significant in any of the age groups, in both olds and news, p>0.05. For elevated TG, the relationship was significant only in those in the old group who were less the 65 years (p≤0.05), but not for those 65 and above in this group, and all the age categories in the new group, p>0.05. All age groups in both the news and olds related significantly with reduced HDL, P<0.05.

**Relationships with sex:** the female gender related significantly, p<0.05, with elevated total cholesterol and elevated LDL in the new group, and reduced HDL in the old group. The relationship was not significant in other groups, p=0.512.

**Relationships with socioeconomic status/family history of hypertension:** similar significant relationships were found with upper and middle SEC in both old and new groups as well as with a positive family history of hypertension in the old group.

**Relationship with duration of hypertension:** this related significantly with elevated total cholesterol, elevated LDL, and elevated TG, but not reduced HDL, p=0.303.

**Relationship with BMI and dysglycaemia:** the relationship with overweight/obesity was significant with elevated total cholesterol (p<0.05 in both old and new), elevated TG/reduced HDL in the old group but not in the new group and with elevated LDL in both groups. Dysglycaemia (prediabetes and diabetes), related significantly with elevated total cholesterol (new and old), elevated LDL/reduced HDL (old only).

## Discussion

The most frequent form of dyslipidemia was reduced high density lipoprotein cholesterol, HDL-C. This is consistent with earlier reports from other parts of Nigeria and elsewhere amongst hypertensive subjects [22-24]. The mean HDL was slightly higher in the new (1.16+0.22) than old (1.14+0.26) group. This may be due to the small sample size of the forma who were also naïve to any drug treatment. Previous studies have suggested that, duration of hypertension may significantly affect the HDL levels which incidentally have been found to be low even in a good proportion of normotensive individuals [23,25]. Pathophysiologically, reduced HDL-C in hypertensive patients is attributable to the effect of the renin angiotensin system on lipid metabolism, with characteristic disturbance of lipid homeostasis [26]. Serum HDL-C level is a dyslipidaemic type thought to contribute to structural and functional changes causing arterial rigidity [27]. Furthermore, studies involving animal models have shown that, reduced HDL-C were associated with significant endothelial dysfunction and compromised peripheral vasodilatation [28] with consequent increase in cardiovascular risks and sequalae. Elevated Low density lipoprotein cholesterol, (LDL-C) was the second most frequent dyslipidaemic type, mainly in the new-group of this cohort of hypertensive subjects. This is consistent with a recent finding by Olamoyegun *et al*. [29] as well as with earlier studies [22-24]. Elevated LDL-C was found in 78.6% of the new group. The old group had a frequency of 48.2%. The mean LDL level was also slightly higher in the new group (3.96+1.04 vs 3.24+0.97). This group is still drug-naïve and may not have had lifestyle education which could have impacted on their lipids profile among others. Elevated LDL-C is thought to be a sequela of severe reduction in hepatic LDL receptor protein, despite normal LDL receptor mRNA and gene translation rate [30]. This may suggest inefficient translation and/or increased LDL receptor protein turnover as a cause of LDL receptor deficiency. Based on the critical role of LDL receptor, acquired LDL receptor-deficiency will result in elevation of plasma LDL-C through impaired LDL clearance and also contribute to elevated total cholesterol [31].

Interestingly, elevated total cholesterol was found in 62.3% of the news as the next most common dslipidaemic type. This pattern of elevated total cholesterol as the third most common lipid abnormality, was reported in an earlier study [32], although our study showed a much higher frequency in both old and new groups than that found in the former study which took place in Ado-Ekiti, in the south-western part of Nigeria. This may be explained by differences in dietary patterns and other life-style factors between Ado-Ekiti and Abuja which is more urbanized. The new group showed a much higher frequency of elevated total cholesterol, 62.3% than the old group, 36.9%. This may be because patients in the new group were treatment-naïve, and were yet to receive life-style related education. The mean of total cholesterol was higher in the new than old (Table 1). Elevated total cholesterol is thought to be due to defective regulatory responses of 3-hydroxy-3- methylglutararyl-CoA reductase and hepatic cholesterol 7α-hydroxylase, which are rate-limiting enzymes in the cholesterol metabolic pathways [23,33]. The fourth commonest dyslipidaemic type in our study was elevated triglycerides. Even though we found higher frequencies, it was consistent with the report from a previous Nigerian study [32]. Elevated triglycerides have been reported in hypertensive subjects in earlier Nigerian studies [23,25,32,33]. This form of dyslipidaemia is attributable to down-regulation of lipoprotein lipase enzymes which are found principally in skeletal muscle, myocardium and adipose tissue [30]. Elevated-triglycerides is a risk factor for cardiovascular diseases, including stroke, because of the increase in chylomicrons and very low-density lipoprotein which are highly thrombogenic, with eventual vascular compromise in hypertensive patients [34]. The consequences of various dyslipidaemic forms are largely cardiovascular diseases [11-13,34]. Elevated total cholesterol levels are known to play key roles in both initiation and progression of atherosclerosis with clinical consequences such as myocardial infarction, stroke, peripheral vascular disease and heart failure, with even a documented curvilinear relationship [11].

The role of elevated LDL-C in the development of CVD cannot be overemphasized, as high levels of LDL-C cause atherosclerosis which have been shown to stabilize and even regress with administration of pharmacologic agents that reduce LDL levels [12]. Low levels of HDL-C have been consistently reported in cardiovascular disease states [13,34]. Certain relationships were observed between the dyslipidaemic types and socio-demographics of the patients, as well as with dysglycaemia (prediabetes and diabetes) and BMI (Table 2). The highest chances for coexisting elevated total cholesterol, (E-TCHOL), in this cohort of patients were observed in those who were; less than 65 years old, (irrespective of the duration of hypertension), female gender, (particularly among the new hypertensive patients), low level of physical activities, positive family history of hypertension, BMI greater than 25kg/m^2^, dysglycaemia and hypertension existing for at least five years. Age has earlier been identified as a correlate of dyslipidaemia in hypertensive patients even in the north central zone of Nigeria, where Abuja is located [35]. Our study showed that hypertensive patients who were aged less than 65 years, largely in the middle-age, were more likely to have associated dyslipidaemic types such as high total cholesterol, high TG and reduced HDL. This is consistent with findings by Adamu *et al*. [35] among hypertensive subjects in Bida, a not so cosmopolitan city, 240km west of Abuja and in same geographic zone. Surprisingly, both the mean total cholesterol levels and the frequency of E-TCHOL, were higher in the new groups than the old in this study. This could be due to the possible lower level of health education among the new patients who were still treatment naïve and may not have been on statins as may have been the case with the previously known hypertensive patients. Surprising too, was the finding of an insignificant relationship between age and high LDL levels in both old and new, and elevated TG in the new group. These are atherogenic forms expected in hypertensive patients especially with advancing age. The smaller sample size in the treatment-naïve new group, may have contributed, while patients in the old-group are treatment-experienced, including statin-use which may have modified the pattern. The patients in this cohort were mainly young and middle-aged, while studies have suggested worsening atherogenic lipids pattern with advancing age [29,36].

**Table 2:** relationships between dyslipidemias forms and variables

Dyslipidaemic form	Variables	p-value (New)	p-value (Old)
Elevated total cholesterol	Age	0.000	0.003
	Sex f	0.011	0.512
	SEC	U 0.337 M 0.343 L 0.797	U 0.245 M 0.245 L 0.183
	Physical activity M	0.013 *	0.074
	Family history of hypertension	0.231	0.015*
	BMI	0.03*	0.034*
	Dysglycaemia	0.000*	0.000*
	Duration of HTN	-	0.005*
Elevated LDL	Age	0.242	0.296
	Sex	0.022*	0.073
	SEC	U 0.221 M 0.251 L 0.923	U 0.001* M 0.001* L 0.000*
	Physical activity	0.501	0.05
	Family history of hypertension	0.069	0.01*
	BMI	0.332	0.067
	Dysglycaemia	0.496	0.000*
	Duration of HTN	-	0.000*
Elevated TG	Age	0.100 (<44 yrs)* 0.097(45-64yr) * 0.218(>65 yrs) *	0.002(<44yrs) * 0.002(45-64yr) * 0.060(>65yrs) *
	Sex	0.370	0.097
	SEC	U 0.238 M 0.237 L 0.148	U 0.005* M 0.005* L 0.82
	Physical activity	0.172	0.243
	Family history of hypertension	0.035*	0.013*
	BMI	0.291	0.001*
	Dysglycaemia	0.370	0.118
	Duration of HTN	-	0.148
Reduced HDL	Age	0.036 (<44yrs) * 0.018(45-64yr) * 0.010 (>65yrs) *	0.000 (<44yrs) * 0.000(45-64 yr) * 0.001 (>65 yrs) *
	Sex	0.108	0.034*
	SEC	U 0.859 M 0.749 L 0.716	U 0.000* M 0.000* L 0.001*
	Physical Activity	0.171	0.001*
	Family history of hypertension	0.355	0.127
	BMI	0.785	0.000*
	Dysglycaemia	0.181	0.005*
	Duration of HTN	-	0.303

Females in the new group were likely to have elevated-TCHOL and elevated LDL, (p=0.011) (P=0.022) respectively, but not females in the old group (p=0.512) (p=0.073) respectively. Females in the old group were likely to have reduced HDL but not those in the new group. No positive relationship existed with elevated TG (Table 2). Previous studies have reported that, female gender was one of the most important predictors of dyslipidaemia in hypertensive patients [37] and this is thought to be an oestrogen-effect [37,38]. Socio-economic class (SEC), showed positive relationship with certain dyslipidaemic types such as hypertryglycaeridaemia in the upper and middle classes, elevated LDL irrespective of class, and reduced HDL, all in the old group. This may be a reflection of failed lifestyle-counselling in these patients who were even treatment-experienced. This is further expressed in their lower physical activity levels which related well with some dyslipidaemic types. This observation is new and inconsistent with findings in previous studies that showed that lower socio-economic class may be associated with higher dyslipidaemic frequencies and hence higher cardiovascular disease risk and sequalae [39,40]. The trend of non-communicable disease vulnerability among the upper SEC in Abuja, was recently suggested in a study by Onyegbutulem *et al*. [41]. Patients in the old group with known family history of hypertension were likely to have elevated TCHOL, elevated LDL and TG, with a possible familial factor. This was not observed in the new group who may not have had a robust history documented over time as with the old patients and may be because of the small size. In our study, dysglycaemia, (assessed with FBG) and adiposity, measured with body mass index BMI, two components of metabolic syndrome [1,5], related positively with dyslipidaemic types in patients with hypertension, particularly long standing.

This is consistent with reports from earlier studies [29,35], although with a higher frequency of abnormal BMI, 51% vs 30.7%. One of the previous studies was conducted in a city in the southwestern part of Nigeria whose socioeconomic indices are lower than that of Abuja [42,43], a city with high occurrence of affluent life-style, atherogenic diet and rising rates of obesity [17,43], mirroring epidemiologic transition [44]. The higher frequency of dysglycaemia in the old group may be attributable to the phenomenon of hypertensive dyslipidemia, insulin resistance [22,45] and possibly the use of certain antihypertensive drugs such as thiazide diuretics, which have been previously reported to increase diabetes risk [46,47]. Even though this association was not tested in this study, 29.9% of the patients had thiazide diuretics for blood pressure control. The possibility of an existing diabetes mellitus in hypertensive patients may also be high [48], as shown, in this study where prediabetes/diabetes were found in 21.6%/7.4% and 26%/0% in old and new groups respectively. Coexistence of these multiple factors increase cardiovascular risk profile, which may likewise, be reduced if they are recognized early and appropriate interventions administered [22,45,49]. While current epidemiologic trends suggest rising incidence of non-communicable diseases in developing economies such as Nigeria´s [50], dyslipidaemias are recognized as major modifiable cardiovascular disease (CVD) risk factors [49] and have been identified as independent risk factors for essential hypertension giving rise to the term 'dyslipideamic hypertension´ [45]. Globally, about 80% of hypertensive persons have metabolic co-morbidities such as obesity, dysglycaemia, hyperisulineamia alongside dyslipidaemias [22]. Such combinations worsen cardiovascular risk.

## Conclusion

Dyslipidemias are very common amongst hypertensive patients in Abuja, the capital city of Nigeria with an already existing high cardiovascular risk profile. The commonest dyslipidaemic type is reduced HDL, followed by elevated LDL, elevated TCHOL and elevated TG. Also, middle age, female gender, middle/high socio-economic class, low levels of physical activity, obesity/overweight, dysglycaemia and long duration of hypertension are related to forms of dyslipidemia in this cohort of hypertensive patients. It is worrisome that, even newly diagnosed hypertensive patients had existing dyslipidemia since it implies high cardiovascular risk which can only be assessed if diagnosed.

**Recommendations:** screening all hypertensive patients for abnormal lipids and appropriate intervention carried out should be the rule. We recommend routine screening of all hypertensive patients for dyslipidemia and lipids lowering drugs recommended accordingly. Educate patients on lifestyle measures to ensure weight control, healthy diet and physical activities.

**Limits/strength:** this was a retrospective study, using secondary data, with limits on the freedom with which its results regarding causal relationships can be interpreted. Hence, prospective longitudinal studies should now be carried out, with larger sample sizes. However, this study has provided sensitization on some existing or emerging trends, adding to the medical literature of Abuja, a strategic and reference city in West Africa.

### What is known about this topic

- Dyslipidemia is common among hypertensive patients, in a pattern of reduced HDL, followed by elevated LDL, elevated triglycerides and total cholesterol;
- Long standing hypertension, advanced age are predictors of dysglycaemia in hypertensive patients.

### What this study adds

- Female gender, middle and high socio-economic classes are the groups with the highest chances of dyslipidemias in the Abuja population;
- Newly diagnosed hypertensive patients in Abuja already have high frequencies of the various dyslipidemic forms.
